# Medicinal Mushroom Extracts from *Hericium coralloides* and *Trametes versicolor* Exert Differential Immunomodulatory Effects on Immune Cells from Older Adults *In Vitro*

**DOI:** 10.3390/nu15092227

**Published:** 2023-05-08

**Authors:** Lily M. Williams, Bronwyn S. Berthon, Isobel L. Stoodley, Evan J. Williams, Lisa G. Wood

**Affiliations:** 1 School of Biomedical Sciences and Pharmacy, College of Health, Medicine and Wellbeing, University of Newcastle, Callaghan, NSW 2308, Australia; lily.williams@newcastle.edu.au (L.M.W.);; 2 Immune Health Research Program, Hunter Medical Research Institute, New Lambton Heights, NSW 2305, Australia

**Keywords:** peripheral blood mononuclear cells, humans, aging

## Abstract

Medicinal mushroom extracts (MMEs) exert immunomodulatory effects on innate immunity. The present study aimed to examine the effect of medicinal mushroom components on *in vitro* immune cell responses to inflammatory stimuli by peripheral blood mononuclear cells (PBMCs) isolated from older adults, where immune function is altered. PBMCs were treated with extracts from *Hericium coralloides* (HC) and *Trametes versicolor* (TV) prior to stimulation with rhinovirus A1 (RVA1), influenza A/H1N1pdm09 (H1N1), lipopolysaccharide (LPS), or house dust mite (HDM) for 48 h. In the presence of virus, type I and II IFN significantly (*p* < 0.05) decreased following treatment with at least one concentration of all extracts compared to the untreated cell controls, along with significant increases in pro-inflammatory cytokines (IL-1β, IL-6, IL-8). In the presence of LPS, extracts from TV reduced IL-1β compared to untreated cells. In the presence of HDM, the concentration of IL-5 and/or IL-13 was significantly decreased with at least one dose of all extracts. MMEs exert differential effects on the release of inflammatory and antiviral mediators *in vitro*. Reduced type 2 cytokine responses to HDM may be beneficial in conditions where allergic inflammation is present, including asthma, allergic rhinitis, and eczema. Further research is needed to examine extracts *in vivo*.

## 1. Introduction

Medicinal mushrooms are considered functional foods because they are rich in bioactive ingredients that are physiologically beneficial. Unlike edible mushrooms normally consumed in the diet, medicinal mushrooms are not consumed whole. Rather, aqueous extracts from both the mycelium (root system) and basidiomes (fruiting bodies) are used as nutraceuticals [1]. Medicinal mushroom extracts (MMEs) have a variety of applications as nutraceuticals due to their known anti-inflammatory, antimicrobial, antioxidant, and immunomodulatory properties [2]. Several mushroom species are considered to have nutraceutical properties, including species under the genus of *Hericium* and *Trametes*. The biological activity of medicinal mushrooms has, in part, been attributed to their polysaccharide content, which consists primarily of β-glucans [3]. Evidence to date demonstrates that polysaccharide components induce immunomodulatory activity on a number of innate immune cells [4]. Polysaccharides from aqueous mushroom extracts induce a pro-inflammatory effect on macrophages (i.e., increasing nitric oxide (NO) synthesis and IL-6 release), while polysaccharides with a high molecular weight or from alkaline extracts demonstrate anti-inflammatory effects (e.g., enhancing IL-10 release). Glucans have been shown to enhance MHC Class II and IL12p40 on dendritic cells, which may enhance type I-mediated adaptive immunity required for anti-viral defense. However, there is growing interest in other bioactive ingredients, including antioxidant compounds (phenolic compounds, flavonoids, carotenoids, and sterols).

*Hericium* (*H.*) *coralloides* (HC), commonly known as Coral Tooth Fungus, is an edible mushroom belonging to the class of *Agaricomycetes*. Extracts from HC contain the bioactive ingredients corallocins, and have been shown to have potent antioxidant activity, increasing superoxide dismutase (SOD), catalase (CAT), and glutathione peroxidase (GSH-Px), and lowering the levels of malondialdehyde (MDA) in animal models [5,6,7]. HC extracts have also displayed antiviral activity, with significant inhibitory activity toward human immunodeficiency virus-1 (HIV-1) reverse transcriptase *in vitro* [8].

*Trametes* (*T.*) *versicolor* (TV), known as Turkey Tail, is another medicinal mushroom known to have immunomodulatory effects. Extracts from TV contain the bioactive components, protein-bound polysaccharide-K (PSK) and polysaccharopeptide (PSP) [2]. *In vitro*, TV has been shown to increase anti-inflammatory cytokines, including interleukin-1 receptor antagonist (IL-1ra) and IL-10, anti-viral cytokines interferon-gamma (IFN-γ) and macrophage inflammatory protein-alpha (MIP-1α), as well as granulocyte-colony stimulating factor (G-CSF) and IL-8 in human peripheral blood mononuclear cell (PBMC) cultures [9]. Further, polysaccharides from TV have been shown to induce B cell activation through nuclear factor kappa B (NFκB) and mitogen-activated protein kinase (MAPK) signaling [10]. Interestingly, TV extracts containing a high phenolic content have been shown to have the greatest antioxidant activity *in vitro* [11]. In human participants, oral administration of TV extract has been shown to increase lymphocyte counts and natural killer (NK) cell function, as well as CD8+ T cells and CD19+ B cells [12].

Immune function declines with increasing age, with a dampening of both adaptive and innate immune responses shown in older adults [13]. Given that MMEs are proposed to improve immune function, older adults are a suitable target population, in whom the effects of these extracts have not been characterized. This study uses an *in vitro* approach to testing the effects of MMEs on immune function in humans. PBMCs were isolated, and the responses to various triggers of inflammation, including viruses (rhinovirus, influenza), bacterial toxins (lipopolysaccharide, LPS), and common allergens (house dust mite, HDM) were examined. Models utilizing a variety of triggers are important in understanding how MMEs may modulate immune cell responses. Viral RNA from influenza A virus and rhinovirus are sensed by pathogen recognition receptors (PRRs), including TLR3 and TLR7 [14,15], while LPS from Gram-negative bacteria activated TLR4 signaling [16]. *Der p I* from HDM activates both TLR4 signaling and protease-activated receptor (PAR)1 and PAR4 [17]. Activation of these different PRRs leads to distinct innate immune cell responses. Commercially available MMEs are available as various preparations, targeting the extracts of water-soluble and -insoluble components. These preparations may include a combination of different solvents, including water, ethanol, and glycerin. The effects of extract solvent on each formulation and the effects of biological responses are unknown. As such, the aim of the present study is to examine the effect of medicinal mushroom (HC, TV) extracts, which utilize various solvents, on the release of inflammatory and anti-viral mediators in PBMCs isolated from healthy, older adults.

## 2. Materials and Methods

### 2.1. Study Design and Population

This was an *in vitro* study conducted using PBMCs isolated from blood samples collected at a single study visit from healthy, older adults (*n* = 15 total: *n* = 8 per experiment). Healthy, non-smoking adults aged 50 years or older were recruited from existing volunteer databases. Exclusion criteria: smoking currently (smoked within the previous 6 months); maintenance use of systemic corticosteroid, immunosuppressive, or antibiotic drugs; unstable cardiac, renal, hypertensive, pulmonary, endocrine, immunologic, or neurologic disorders; acute or terminal illness, human immunodeficiency virus (HIV) or active cancer. This study was conducted at the Hunter Medical Research Institute (HMRI) Newcastle, Australia, in accordance with the Declaration of Helsinki and ICH GCP standards, with approval from the Hunter New England Human Research Ethics Committee (2021/ETH11012) and the University of Newcastle Human Research Ethics Committee (H-2021-0348). All participants provided written informed consent prior to study entry.

### 2.2. Blood Processing and Peripheral Blood Mononuclear Cell Isolation

Blood was collected via venipuncture into 9 mL ethylenediamine-tetra acetic acid (EDTA) tubes following an overnight (≥12 h) fast. Blood samples were centrifuged at ~1700× *g* for 10 min at 22 °C, plasma was removed from the top phase with the remaining cell fraction used to isolate PBMCs. PBMCs were isolated via density gradient centrifugation using LymphoprepTM (STEMCELL Technologies, Vancouver, BC, Canada) and 50 mL SepMate™ tubes (STEMCELL Technologies, Vancouver, BC, Canada), as per the manufacturer’s instructions. Isolated PBMCs were counted and assessed for viability via staining with trypan blue 0.4%, then resuspended in Roswell Park Memorial Institute (RPMI) media (RPMI-1640; Sigma-Aldrich, St. Louis, MO, USA) with 10% fetal bovine serum (FBS) and plated in 24-well tissue culture plates (Sigma-Aldrich, United States) at a final density of 2 × 10^6^ live cells/mL.

### 2.3. In Vitro Cell Culture of Peripheral Blood Mononuclear Cells

Stock solutions of eight different MMEs (Rembiotics Pty Ltd., Ormeau, Australia) from *H. coralloides* (HC) and *T. versicolor* (TV), species which are native to Australia, were provided by Rembiotics Pty Ltd., Byron Bay, Australia at a concentration of 500 mg/mL. Four different solvents were used to generate extracts from HC and TV, including water–ethanol, with (KP) and without (WE) Kakadu Plum, water–glycerin (WG), and water–glycerin liposomal (WGL).

Plated PBMCs were treated with each of the eight extracts, separately, at 1 mg/mL and 10 mg/mL for 3 h. Following treatment, PBMCs were stimulated, separately, with or without 1 multiplicity of infection (MOI) rhinovirus A1 (RVA1), 0.1 MOI influenza A/H1N1pdm09 (H1N1) [18], 5 ng/mL lipopolysaccharide (LPS, *E. coli*) [19,20], and 100 μg/mL house dust mite (HDM, *D. pteronyssinus*) [21,22] for a total of 48 h (35 °C, 5% CO_2_). Extract treatment concentrations were determined using dose–response curves for IL-6 following H1N1 and LPS stimulation, and IL-5 for HDM stimulation, respectively using PBMCs from healthy controls. Cell culture controls included no treatment, as well as ethanol and glycerin controls with equal concentration of ethanol and glycerin in 10 mg/mL treatments. At 48 h, cell suspensions were spun at ~550× *g* for 5 min at 22 °C, and cell-free supernatant was stored at −80 °C for analysis.

### 2.4. LEGENDplex^TM^ Assay

The concentration of cytokines in cell-free supernatant was determined via the LEGENDplex^TM^ assay (BioLegend, San Diego, CA, USA) using the Inflammation 1 (Catalog # 740809: RVA1, H1N1, LPS) and T Helper 2 (Catalog # 741034: HDM) panels, as per manufacturer’s instructions. The concentration of IL-1β, IL-6, IL-8, IL-10, IFN-α2, and IFN-γ were measured in RVA1 and H1N1-stimulated cell cultures. The concentration of IL-1β, IL-6, IL-8, IL-10, and TNF-α were measured in LPS-stimulated cell cultures. The concentration of IL-4, IL-5, IL-13, IL-6, IL-10 and TNF-α were measured in HDM-stimulated cell cultures.

### 2.5. Statistical Analysis

Data were analyzed using GraphPad Prism 9 (GraphPad Software, La Jolla, CA, USA). Normality was assessed using Shapiro–Wilk tests. Parametric data are reported as mean ± standard deviation (SD), and non-parametric data are reported as median (1st quartile, 3rd quartile). The difference between the concentration of inflammatory mediators in cell culture supernatants from treated and untreated cells was analyzed using one-way analysis of variance (ANOVA) for parametric data, or Friedman Test for non-parametric data. Appropriate post hoc testing was used to compare treated cells with the untreated cell control. Significance was accepted if *p* < 0.05.

## 3. Results

### 3.1. Subject Characteristics

Characteristics of the 15 subjects who participated in the study are summarized in Table 1. The average age of participants was 69 years, and 60% of subjects were female. The average BMI was ~29 kg/m^2^, indicating an overweight population. Almost half of subjects (*n* = 7, 47%) were ex-smokers, with mean pack years of 13.6.

### 3.2. Dose–Response Relationships between Mushroom Extract Concentration and Inflammatory Mediator Release across Models of Stimulation

**Figure 1:**
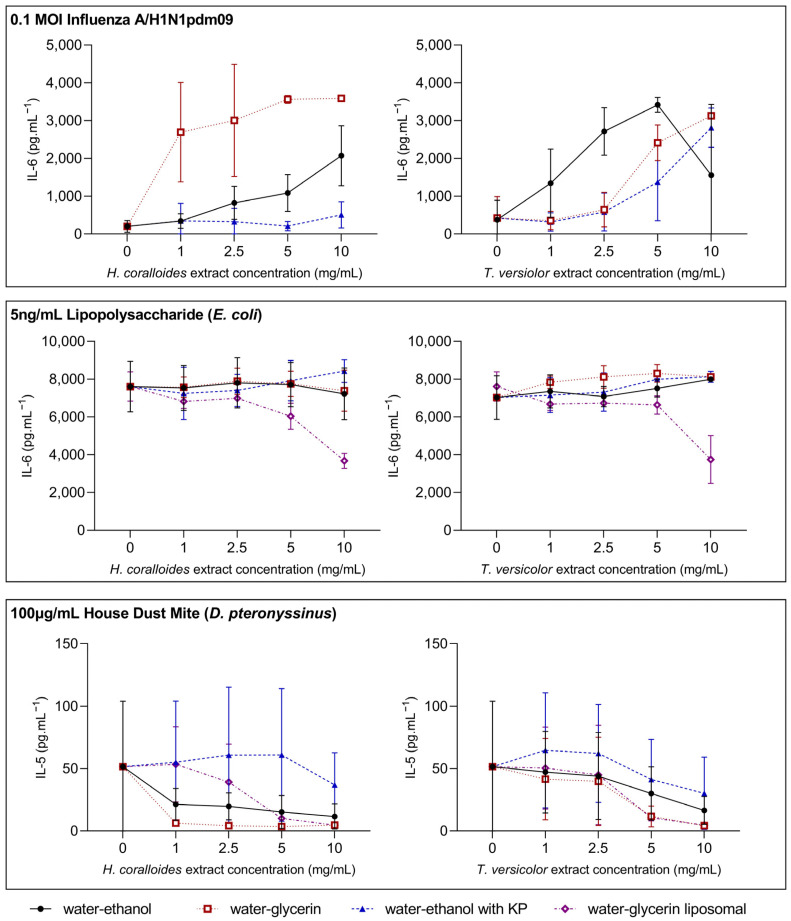
Dose–response relationships between mushroom extract concentration and inflammatory mediator release following stimulation. Peripheral blood mononuclear cells (PBMCs) were isolated from whole blood collected from healthy volunteers (*n* = 3–5) and seeded at a density of 2 × 10^6^ cells/mL. Cells were treated with 1, 2.5, 5, and 10 mg/mL *Hericium* (*H.*) *coralloides* and *Trametes* (*T.*) *versicolor* extracts (water–ethanol without Kakadu Plum (KP), water–ethanol with KP; water–glycerin, water–glycerin liposomal) for 3 h, and then stimulated with 0.1 MOI influenza A/H1N1pdm09 (top panel, *n* = 5), 5 ng/mL lipopolysaccharide (*E. coli*) (center panel, *n* = 5), or 100 µg/mL house dust mite (*D. pteronyssinus*) (bottom panel, *n* = 3) for a total of 48 h at 35 °C, 5% CO_2_. The concentration of interleukin (IL)-6 (top and center panel) and IL-5 (bottom panel) were measured via DuoSet ELISA. Data are displayed as mean ± standard deviation.

The effect of increasing MME concentrations on the release of inflammatory mediators in PBMCs was examined in healthy adults aged ≤50 years, *n* = 3–5, in cells pre-treated with 1, 2.5, 5, or 10 mg/mL of HC and TV (water–ethanol without Kakadu Plum (KP); water–ethanol with KP; water–glycerin; water–glycerin liposomal), extract concentrations. IL-6 was measured in models of virus infection (influenza A/H1N1, H1N1) and bacterial infection (lipopolysaccharide, LPS), while IL-5 was measured in an allergen exposure (house dust mite, HDM) model in cell culture supernatants (Figure 1).

H1N1 stimulation did not affect IL-6 levels, while mushroom extract treatment generally led to dose-dependent increases in IL-6, with peak responses for most extracts occurring at 10 mg/mL (Figure 1). In contrast, LPS stimulation increased IL-6, and there did not appear to be a dose-dependent relationship between mushroom extract concentration and IL-6 levels following LPS stimulation (Figure 1). At a 10 mg/mL concentration for water–glycerin liposomal extracts, there was a decrease in LPS-induced IL-6. Following HDM stimulation, IL-5 increases, while most extracts display a dose-dependent decrease in IL-5 (Figure 1). The exception to this relationship was the water–ethanol extract from HC containing Kakadu Plum (HCKP). Based on these data, extract concentrations of 1 mg/mL and 10 mg/mL were selected for further cell culture model experiments.

### 3.3. Mushroom Extracts Reduce Type I and II IFN, While Increasing Pro-Inflammatory Mediator Release to Rhinovirus and Influenza Stimulation

**Figure 2:**
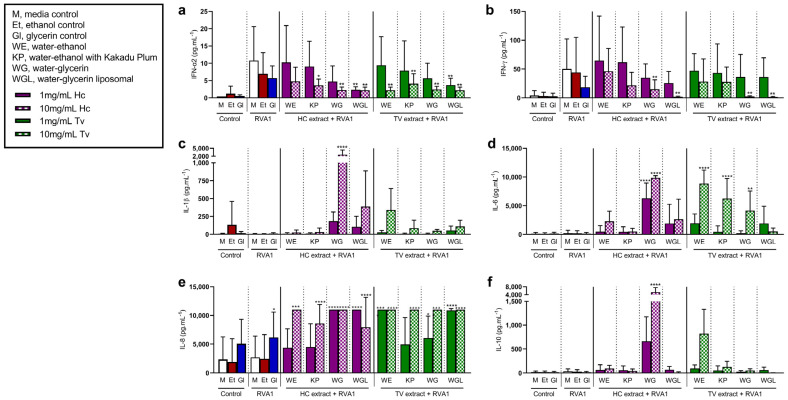
Effects of *Hericium coralloides* and *Trametes versicolor* extracts on immune cell responses to rhinovirus A1 (RVA1) stimulation. Peripheral blood mononuclear cells (PBMCs) were isolated from whole blood (*n* = 8) and seeded at a density of 2 × 10^6^ cells/mL. Cells were treated with 1 (solid color) or 10 mg/mL (check pattern) mushroom extracts (HC, *Hericium coralloides*; TV, *Trametes versicolor*) for 3 h, and then stimulated with 1 MOI RVA1 for a total of 48 h at 35 °C, 5% CO_2_. The concentration of (**a**) interferon (IFN)-α2, (**b**) IFN-γ, (**c**) interleukin (IL)-1β, (**d**) IL-6, (**e**) IL-8, and (**f**) IL-10 were measured via the LEGENDplex^TM^ assay. Data are displayed as mean with standard deviation, analyzed using one-way ANOVA with Holm–Sidak multiple comparisons where appropriate. * *p* < 0.05, ** *p* < 0.01, *** *p* < 0.001, **** *p* < 0.0001, compared to RVA1 media control. M, media control; E, ethanol control; G, glycerin control; WE, water–ethanol; KP, water–ethanol with Kakadu Plum; WG, water–glycerin; WGL, water–glycerin liposomal.

**Figure 3:**
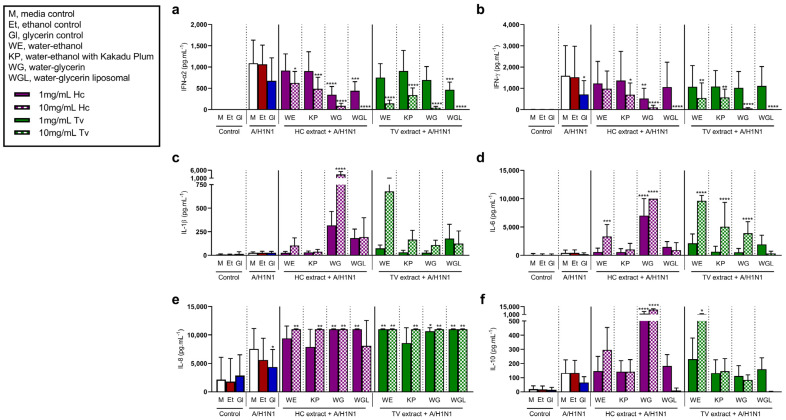
Effects of *Hericium coralloides* and *Trametes versicolor* extracts on immune cell responses to influenza A/H1N1pdm09 (H1N1) stimulation. Peripheral blood mononuclear cells (PBMCs) were isolated from whole blood (*n* = 8) and seeded at a density of 2 × 10^6^ cells/mL. Cells were treated with 1 (solid color) or 10 mg/mL (check pattern) mushroom extracts (HC, *Hericium coralloides*; TV, *Trametes versicolor*) for 3 h, and then stimulated with 0.1 MOI H1N1 for a total of 48 h at 35 °C, 5% CO_2_. The concentration of (**a**) interferon (IFN)-α2, (**b**) IFN-γ, (**c**) interleukin (IL)-1β, (**d**) IL-6, (**e**) IL-8, and (**f**) IL-10 were measured via the LEGENDplex^TM^ assay. Data are displayed as mean with standard deviation, analyzed using one-way ANOVA with Holm–Sidak multiple comparisons where appropriate. * *p* < 0.05, ** *p* < 0.01, *** *p* < 0.001, **** *p* < 0.0001, compared to H1N1 media control. M, media control; Et, ethanol control; Gl, glycerin control; WE, water–ethanol; KP, water–ethanol with Kakadu Plum; WG, water–glycerin; WGL, water–glycerin liposomal.

To examine the effects of HC and TV species in different extract solvents on innate immune cell responses to viruses, pre-treated PBMCs from older adults were stimulated with either rhinovirus A1 (RVA1) (Figure 2) or influenza A/H1N1pdm09 (H1N1) (Figure 3). In cell culture supernatants from RVA1- and H1N1-stimulated PBMCs, the concentrations of interferons (IFN-α2 and IFN-γ), pro-inflammatory cytokines (IL-1β, IL-6, and IL-8), and anti-inflammatory cytokines (IL-10) were measured.

In the presence of RVA1 and H1N1, type I (IFN-α2) and II (IFN-γ) IFNs were elevated compared to media controls. RVA1 induced. Type I IFN (IFN-α2) in the presence of RVA1 was reduced with 10 mg/mL of all extracts, except for HC water–ethanol extract (HCWE) (Figure 2a). There was also a decrease in RVA1-induced IFN-α2 with 1 mg/mL water–glycerin liposomal (WGL) extracts from both HC (*p* = 0.003) and TV (*p* = 0.003). The concentration of IFN-γ decreased with 10 mg/mL HCWG, HCWGL, TVWG, and TVWGL (Figure 2b). In contrast, in RVA1 models, IL-6 increased with both 1 and 10 mg/mL HCWG, as well as 10 mg/mL of all TV extracts except WGL (WE, KP, and WG) (Figure 2d). IL-8 increased with at least one dose of all mushroom extracts, following RVA1 compared to RVA1 media controls (Figure 2e). Following RVA1 stimulation, both IL-1β (Figure 2c) and IL-10 (Figure 2f) were increased with 10 mg/mL HCWG compared to untreated cells.

Like RVA1 stimulation, H1N1 increased IFN-α2 following stimulation, and IFN-α2 levels were significantly lower in cells pre-treated with at least one concentration of each extract (*p* < 0.05) (Figure 3a). These effects were similar for IFN-γ (Figure 3b) in the presence of H1N1, compared to untreated cells. Further, in the H1N1 model, the release of IL-6 increased with HCWE (10 mg/mL), HCWG (1 mg/mL, 10 mg/mL), TVWE (10 mg/mL), TVKP (10 mg/mL), and TVWG (10 mg/mL) (Figure 3d). Similarly, IL-8 increased following H1N1 stimulation with at least one concentration of each extract compared to untreated cells (Figure 3e). The concentration of IL-1β induced by H1N1 stimulation increased with 10 mg/mL HCWG treatment, compared to untreated cells (Figure 3c), while H1N1-induced IL-10 increased with HCWG (1 mg/mL, 10 mg/mL) and TVWE (10 mg/mL, *p* = 0.033) (Figure 3f).

### 3.4. Interleukin-1β Is Decreased with Mushroom Extracts from Trametes versicolor in the Presence of Lipopolysaccharide

**Figure 4:**
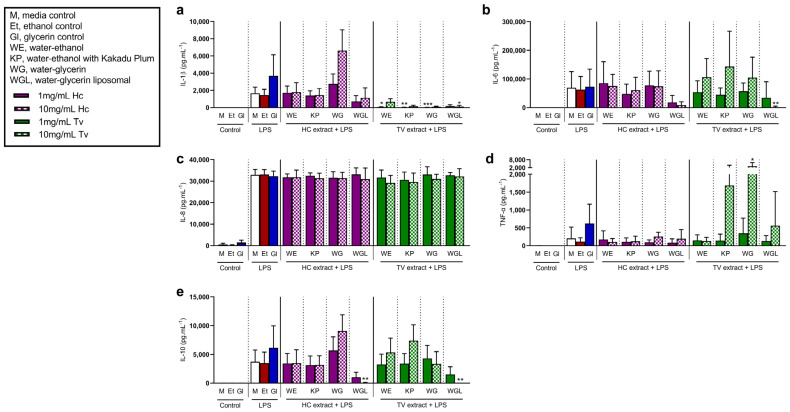
Effects of *Hericium coralloides* and *Trametes versicolor* extracts on immune cell responses to lipopolysaccharide (LPS) stimulation. Peripheral blood mononuclear cells (PBMCs) were isolated from whole blood (*n* = 8) and seeded at a density of 2 × 10^6^ cells/mL. Cells were treated with 1 (solid color) or 10 mg/mL (check pattern) mushroom extracts (HC, *Hericium coralloides*; TV, *Trametes versicolor*) for 3 h, and then stimulated with 5 ng/mL LPS (*E. coli*) for a total of 48 h at 35 °C, 5% CO_2_. The concentration of (**a**) interleukin (IL)-1β, (**b**) IL-6, (**c**) IL-8, (**d**) tumor necrosis factor (TNF)-α, and (**e**) IL-10 were measured via the LEGENDplex^TM^ assay. Data are displayed as mean with standard deviation, analyzed using one-way ANOVA with Holm–Sidak multiple comparisons where appropriate. * *p* < 0.05, ** *p* < 0.01, *** *p* < 0.001, compared to LPS media control. M, media control; E, ethanol control; G, glycerin control; WE, water–ethanol; KP, water–ethanol with Kakadu Plum; WG, water–glycerin; WGL, water–glycerin liposomal.

To examine the effects of MME pre-treatment on innate immune cell responses to inflammatory stimuli, PBMCs from older adults were stimulated with lipopolysaccharide (LPS) (Figure 4), a Toll-like receptor 4 (TLR) agonist. Cell culture supernatants from LPS-stimulated PBMCs were analyzed for the concentrations of pro-inflammatory (IL-1β, IL-6, IL-8, and TNF-α) and anti-inflammatory (IL-10) cytokines.

The concentration of LPS-induced IL-1β was reduced with 1 mg/mL TVWE (*p* = 0.021), TVKP (*p* = 0.001), and TVWG (*p* < 0.001) treatment, compared to untreated LPS control cells (Figure 4a). In the presence of 10 mg/mL TVWGL, LPS-induced IL-6 (*p* = 0.005, Figure 4b) and IL-10 (*p* = 0.006, Figure 4e) were reduced, while 10 mg/mL HCWGL decreased LPS-induced IL-10 only (*p* = 0.008). There was also an increase in TNF-α in the presence of LPS with 10 mg/mL TVWG (*p* = 0.016, Figure 4d). LPS stimulation increased IL-8, while extract pre-treatment had no effect on LPS-stimulated cells compared to untreated cells (Figure 4c). HCWGL (10 mg/mL) pre-treatment reduced IL-10 (*p* = 0.009).

### 3.5. Type 2 Cytokines Induced by House Dust Mite Are Suppressed by Treatment with Mushroom Extracts

**Figure 5:**
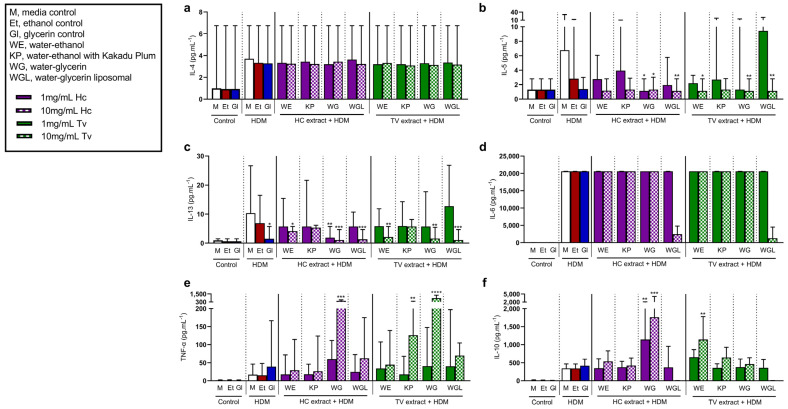
Effects of *Hericium coralloides* and *Trametes versicolor* extracts on immune cell responses to house dust mite (HDM) stimulation. Peripheral blood mononuclear cells (PBMCs) were isolated from whole blood (*n* = 8) and seeded at a density of 2 × 10^6^ cells/mL. Cells were treated with 1 (solid color) or 10 mg/mL (check pattern) mushroom extracts (HC, *Hericium coralloides*; TV, *Trametes versicolor*) for 3 h, and then stimulated with 100 μg/mL HDM (*D. pteronyssinus*) for a total of 48 h at 35 °C, 5% CO_2_. The concentrations of (**a**) interleukin (IL)-4, (**b**) IL-5, (**c**) IL-13, (**d**) IL-6, (**e**) tumor necrosis factor (TNF)-α, and (**f**) IL-10 were measured via the LEGENDplex^TM^ assay. Data are displayed as medians with an interquartile range, analyzed using the Friedman Test with Dunn’s multiple comparisons where appropriate. * *p* < 0.05, ** *p* < 0.01, *** *p* < 0.001, **** *p* < 0.0001, compared to HDM media control. M, media control; E, ethanol control; G, glycerin control; WE, water–ethanol; KP, water–ethanol with Kakadu Plum; WG, water–glycerin; WGL, water–glycerin liposomal.

To examine the effects of MMEs on type 2 responses to common allergens, PBMCs from older adults were stimulated with house dust mite (HDM) extract from *D. pteronyssinus* (Figure 5). The HDM extract contains the common aeroallergen *Der p I*, which induces proliferative responses from Th2 cells [23], leading to the release of inflammatory mediators, including type 2 cytokines (IL-4, -5, and -13).

HDM induced the release of type 2 cytokines (IL-4, -5, and -13), pro-inflammatory cytokines (IL-6, IL-8, and TNF-α), and anti-inflammatory cytokine (IL-10) by PBMCs. Following HDM exposure, there was no significant difference in IL-4 (Figure 5a) or IL-6 (Figure 5d) following any extract treatment compared to untreated cells. The concentration of HDM-induced IL-5 decreased with 10 mg/mL HCWG, HCWGL, TVWE, TVWG, and TVWGL, compared to untreated cells (Figure 5b). Additionally, HDM-induced IL-5 decreased with 1 mg/mL HCWG treatment. Further, at least one dose of all treatments, except KP extracts, led to reduced HDM-induced IL-13 compared to untreated cells (Figure 5c). In contrast, HDM-induced TNF-α increased with 10 mg/mL HCWG, TVKP, and TVWG treatments compared to untreated cells (Figure 5e). Interestingly, in the presence of HDM, IL-10 increased with both 1 and 10 mg/mL HCWG, as well as 10 mg/mL TVWE (Figure 5f).

## 4. Discussion

The present study aimed to examine the effects of *in vitro* treatment of human PBMC with extracts from medicinal mushrooms, HC and TV, on the release of mediators following stimulation with respiratory viruses (RVA1, H1N1), bacterial endotoxin (LPS), and common aeroallergen (HDM) in a population of healthy, older adults. In the presence of viruses, the overall observation was that MMEs reduced type I and II IFN coupled with enhanced inflammatory responses. Interestingly, following HDM exposure, the release of type 2 cytokines (IL-5 and IL-13) decreased with treatment. For HC water–glycerin and TV water–ethanol extracts, this was coupled with an increase in HDM-induced IL-10.

The cell culture models presented in this paper examined the effects of MMEs on early innate immune cell responses to both influenza virus and rhinovirus, demonstrating deficient virus-induced IFN-α and IFN-γ at 48 h, with some extracts demonstrating this combined with elevated IL-6. These data are consistent with previous research demonstrating aqueous extracts from the mycelium of TV decrease IFN-γ from unstimulated PBMC cultures in a dose-dependent manner [9], suggesting a reduced Th1 response required for anti-viral immunity, as robust T-helper 1 (Th1) responses to virus infection, consisting of IFN-γ-secreting T cells specific to the virus and cytotoxic T cells, is required for the clearance of infection [24,25].

The present study found *in vitro* treatment of PBMCs with MMEs elevated IL-6 in the presence of both influenza and rhinovirus. Previous research indicates (1,3)-β-glucans present in medicinal mushrooms exert pro-inflammatory effects that are, in part, dependent on NLRP3 inflammasome activation and dectin-1/Syk signaling [26]. In addition, polysaccharides from *Agaricus blazei Murill* (AbM) have been shown to signal via TLR2, inducing pro-inflammatory cytokines, including IL-6 and TNF-α in monocytes [27]. This elevated pro-inflammatory response may have negative physiological consequences, as recent evidence implicates an elevated IL-6 response coupled with reduced IFN-γ as a predictor for severe disease in cases of viral respiratory infection [28]. These data support previous *in vitro* studies demonstrating extracts from AbM induce dose-dependent increases in IL-1β, IL-6, IL-8, and TNF-α from monocytes [29], while extracts from the AbM basidiomes lead to increased TNF-α and IL-8 from macrophages [30].

Elevated pro-inflammatory responses *in vitro*, however, may not translate to negative physiological effects *in vivo*. Data from a previous study found that while in vitro treatment with MMEs elevated concentrations of IL-6, IL-8, and TNF-α in a dose-dependent manner, *in vivo* consumption of extracts led to decreased IL-1β, IL-6, and TNF-α after 12 d of supplementation [31]. The study in question hypothesized this may be due to the movement of antioxidant components across the intestinal mucosa, while absorption of the larger and more complex β-glucan structures was impaired. Further, the beneficial effects of the β-glucan content *in vivo* may be partially attributed to interaction with intestinal microbiota, with extracts from *H. erinaceus* shown to reduce TNF-α-positive cells in colonic mucosa and exert beneficial effects on gut bacteria in an animal model of inflammatory bowel disease [32].

In the presence of LPS, the authors observed an increase in both pro-inflammatory (IL-1β, IL-6, IL-8, and TNF-α) and anti-inflammatory (IL-10) cytokines. LPS is a ligand for Toll-like receptor 4 (TLR4), a receptor expressed on innate immune cells that may be activated by LPS from Gram-negative bacteria [33]. Treatment with TV extracts decreased IL-1β in the presence of LPS stimulation, which may be beneficial in an older population. Aging is associated with elevated inflammation, with studies demonstrating that age-related changes in NFκB signaling upregulate the expression of pro-inflammatory genes (IL-1β, IL-6) [34]. Further, data suggest sensitivity to LPS increases with age, demonstrating greater upregulation of the NLRP3 inflammasome and IL-1β in aged rats versus younger controls [35]. Validation of these observations is required in humans.

House dust mite (HDM) is a common aeroallergen, causing clinical symptoms in subjects with atopic conditions, including asthma, allergic rhinitis, and dermatitis. *Der p I* is one of the major mite allergens isolated from *D. pteronyssinus*, which causes proliferative T cell responses, mainly from T-helper 2 (Th2) cells, in subjects with sensitivity to the allergen [23], resulting in the release of type 2 cytokines (IL-4, IL-5, and IL-13). These cytokines are key in the allergic response. In the present study, the authors examined the effects of MMEs on inflammatory mediator release by PBMCs following HDM stimulation. All extracts, except for water–ethanol extracts containing KP, reduced IL-5 and/or IL-13 from cells compared to untreated cells, suggesting a beneficial impact on type 2 inflammatory responses. Previous data show that consumption of AbM extracts for 3 weeks reduced circulating IL-5 concentrations in patients with ulcerative colitis compared to baseline [36]. Further, an extract from AbM mycelia has been shown to reduce specific IgE and basophil sensitivity in a cohort of subjects with IgE-confirmed birch pollen allergy and asthma [37]. In contrast to the discrepancy between other pro-inflammatory cytokines, the effects on type 2 cytokines appear consistent between *in vitro* and *in vivo* administration. More research is required to examine the benefit of *in vivo* MME administration in subjects with allergic disease.

In contrast to suppressive effects on type 2 cytokines, treatment with TVKP and both TV and VC water–glycerin extracts was associated with increased TNF-α following HDM stimulation. TNF-α is pleiotropic cytokine produced by macrophages and mast cells and has been identified as a key mediator in the development of type 2 responses to aeroallergens [38,39], such as HDM. However, as previously noted, whether these changes would occur in vivo is unclear and warrants further investigation.

The present study has several strengths, including examining two concentrations across a range of extract solvents from both HC and TV in experimental models with three different stimulatory agents to investigate the effects of extracts across multiple inflammatory pathways, and comprehensive readouts with a minimum of five cytokines measured per model with appropriate controls. However, this study also had limitations. Foremost, the data from the literature indicate variation in cell culture responses depending on whether immune cells are treated with extracts *in vitro* or exposed *in vivo* with extract consumption followed by ex vivo stimulation. Further, it is unclear what components from extracts are absorbed into circulation, and which components may act locally within the gastrointestinal tract. Therefore, *in vitro* treatment of PBMCs with MMEs may expose cells to MME components that may not be present in circulation, for example, β-glucans. Commercially available MMEs are often consumed as whole extracts, not as individual components. However, there is considerable variety in the molecular weight, branching, and chemical composition of polysaccharides found in medicinal mushrooms and their extracts [4]. As such, future studies may perform analyses on extracts and examine the effects of individual mushroom components on immune function. Further, in the present study, a pre-treatment time of 3 h was implemented. However, as some MME components may take longer to induce immunomodulatory activity, future research may wish to investigate longer treatment periods. Finally, older healthy subjects were included due to evidence demonstrating suppressed innate and adaptive immune function [13]. Though younger healthy controls were not included, it is unknown whether the results are generalizable to younger adult populations.

## 5. Conclusions

Data from the present study suggest mushroom extracts from *H. coralloides* and *T. versicolor* modify *in vitro* inflammatory mediator release in PBMCs from older adults following stimulation. Data from virus-stimulated cell cultures indicate pro-inflammatory effects of some extracts, which may indicate enhanced immune cell activation. However, the increase in pro-inflammatory mediators coupled with reduced type I and II IFN levels indicate negative treatment effects. Treatment with TV extracts may play an anti-inflammatory role in response to bacterial infection. For both mushroom species, water–ethanol and water–glycerin extracts reduced type 2 responses, and increased IL-10 following HDM stimulation. This may be beneficial in the context of allergy, where increased type 2 responses drive diseases and conditions such as asthma, allergic rhinitis, and eczema. Further studies are warranted to understand the physiological consequences of these observations.

## Figures and Tables

**Table 1:** Subject characteristics.

N	15
Age, years (range)	68.7 ± 9.5
Weight, kg	82.2 ± 16.6
BMI **^1^**, kg/m^2^	28.87 ± 5.05
Male, *n* (%)	6 (40)
Female, *n* (%)	9 (60)
Ex-smokers, *n* (%)	7 (47)
Smoking history, pack years	13.6 ± 7.9

^1^ BMI, body mass index. All continuous data are normally distributed and presented as mean ± standard deviation (SD).

## Data Availability

The data presented in this study are available on reasonable request from the corresponding author.
